# Essential Trace Elements in Scalp Hair of Residents across the Caspian Oil and Gas Region of Kazakhstan

**DOI:** 10.3390/toxics10070364

**Published:** 2022-06-30

**Authors:** Gulmira Umarova, Gulnara Batyrova, Zhenisgul Tlegenova, Victoria Kononets, Saule Balmagambetova, Yeskendir Umarov, Inkara Yessengaliyeva, Arstan Mamyrbayev

**Affiliations:** 1 Department of Evidence-Based Medicine and Scientific Management, West Kazakhstan Marat Ospanov Medical University, 68 Maresyev Street, Aktobe 030019, Kazakhstan; gulmira.um80@gmail.com; 2 Department of Laboratory and Visual Diagnostics, West Kazakhstan Marat Ospanov Medical University, 68 Maresyev Street, Aktobe 030019, Kazakhstan; 3 Department of Internal Diseases No. 2, West Kazakhstan Marat Ospanov Medical University, 68 Maresyev Street, Aktobe 030019, Kazakhstan; zhenisgultlegenova@yandex.kz; 4 Department of Natural Sciences, West Kazakhstan Marat Ospanov Medical University, 68 Maresyev Street, Aktobe 030019, Kazakhstan; micropaleontolog@yandex.kz (V.K.); eskendir.um@gmail.com (Y.U.); 5 Department of Oncology, West Kazakhstan Marat Ospanov Medical University, 68 Maresyev Street, Aktobe 030019, Kazakhstan; sau3567@gmail.com; 6 Department of General Medical Practice No.1, West Kazakhstan Marat Ospanov Medical University, 68 Maresyev Street, Aktobe 030019, Kazakhstan; esengalievainkara@gmail.com; 7 Department of Hygienic Disciplines with Occupational Disease, West Kazakhstan Marat Ospanov Medical University, 68 Maresyev Street, Aktobe 030019, Kazakhstan; mamyrbaevarstan3@gmail.com

**Keywords:** Caspian coastline, Kazakhstan, mass spectrometry, oil-producing, trace elements

## Abstract

Most of the country’s oil and gas fields are situated in West Kazakhstan, mainly on the Caspian Sea coast, causing significant damage to the local environment and contributing to an imbalance in the trace element composition of the human body. The study is aimed to evaluate the relationship between the concentration of essential trace elements in scalp hair of the western Kazakhstan adult population and the remoteness of their residence from oil and gas fields. The concentration of essential trace elements (Co, Cu, Fe, I, Mn, Se, Zn) in the hair of 850 individuals aged 18–60 years was determined by inductively coupled plasma mass spectrometry. In residents of settlements located at a distance of >110 km from oil and gas fields, the concentration of Cu and I in hair was significantly higher than in those closer to 110 km (*p* < 0.001). The content of Cu and I were associated with the distance to oil and gas fields (0.072 (95% CI: 0.050; 0.094)) and (0.121 (95% CI: 0.058; 0.185)), respectively. We detected a significant imbalance in the distribution of some essential trace elements in residents’ scalp hair from the Caspian region of western Kazakhstan, living near oil and gas fields. The concentrations of Cu and I were significantly interrelated with the distance to oil and gas fields. The level of copper in the hair of both inhabitants of the area most remote from oil and gas facilities and the entire population of western Kazakhstan as a whole remains significantly low. The data obtained provide evidence of the possible impact of pollutants generated by the oil and gas facilities on a shortage of essential trace elements and associated subsequent health risks.

## 1. Introduction

The Republic of Kazakhstan (RK) ranks 8th in the world regarding proven oil reserves but 84th according to the Environmental Performance Index and 137th in the Life Expectancy Index [1]. Despite significant investments in public health and priority reforms to make health services more accessible and efficient, Kazakhstan lags behind high-income countries on health indicators [2]. 

Kazakhstan’s health care system faces challenges associated with unique geospatial, geopolitical, and economic realities that hinder innovations and lead to environmental pollution. The main oil and gas fields are located in the country’s West, and their intensive development has led to significant environmental degradation in this region. By-products of exploitation of oil and gas facilities, such as heavy metals, benzene, toluene, polycyclic aromatic hydrocarbons, and other chemicals, are found in high concentrations in air, water, and soil [3]. The total concentration of petroleum hydrocarbons in underground waters is four times higher than the World Health Organization (WHO) standards [4]. An active change in the chemical and physicochemical properties of the soil is ongoing, and the hydrological regime of these terrains is disturbed [5]. Oil and gas proceedings often lead to heavy metals and other pollutants released into the environment [6]. Researchers reported high health risks associated with exposure to heavy metals in regions of oil and gas fields, where the excess heavy metals released could cause an imbalance in the trace element composition of the human body [7,8,9]. Living in close proximity to petrochemical industry complexes, including oil refineries, is associated with a high level of adverse health effects [10,11]. Some studies report the prevalence of asthma and other respiratory diseases, also diseases of the cardiovascular system among children and adults living in the vicinity of petrochemical complexes [12,13], as well as an increase in adverse effects on pregnancy and childbirth outcomes [11]. Domingo et al. stated that there is an increase in cancer mortality and the incidence of certain types of cancer, especially leukemia and other hematological malignancies among the population living in the area of oil production and processing [10,11]. The researchers describe the change in the concentration of heavy metals belonging to both the toxic group and the essential and conditionally essential groups in the regions of industrial and petrochemical pollution. Moreover, it is stated that the concentration of metals and metalloids formed mainly as a result of anthropogenic activity in the environment has increased significantly [14,15].

The concentration of heavy metals is estimated both in environmental objects—soil, water, air, and in biological objects, including in the human body. Rovira et al. over the past two decades have organized a number of studies aimed at assessing the environmental impact of air pollutants potentially emitted by the petrochemical industrial complex in Tarragona, Spain, including heavy metals [6]. Also, monitoring of the content of heavy metals in the soil, including essential Cu, Fe, Mn, and Zn, conducted in the southwestern part of Louisiana, USA, revealed an increase in their concentrations in the industrial petrochemical region, compared with the non-industrial region [16]. According to Relić et al., there is an increase in the content of Cu and Zn in the soil in areas of petrochemical production in comparison with unpolluted areas, however, this pattern has not been established for the essential elements Co, Mn, and Se [17]. A number of studies report an increase in the concentration of a number of metals and metalloids in drill cuttings during oil production operations at sea. In western Kazakhstan, a significant number of fields, including the largest Kashagan field, are being developed by offshore drilling. The polluting effect of drill cuttings causes an increase in the concentration of essential elements Cu [18,19,20], Fe, and Zn [19,20] in bottom sediments.

In a study by Varrica et al. conducted in the area of contamination by petrochemical production, an assessment of the profile of metals in the hair of adolescents living in this zone showed an increase in the concentration of Zn and Mn [21]. In an earlier paper by Varrica et al., the content of metals and metalloids in the hair of children living in various geochemical conditions in southwestern Sardinia was analyzed. The hair of children living in areas of intensive mining activity showed higher concentrations of most of the metals and metalloids included in the study, including the essential trace element Zn [22]. There was an increase in the concentration of Mn in the urine and hair of pregnant women living in Northeastern British Columbia (Canada) in the area of intensive exploitation of natural gas by hydraulic fracturing [23]. Skalny et al. report an increase in the essential elements Fe, Co, and I in the hair of petrochemical plant workers engaged in bitumen production [24]. Also, in the oldest work of Moon et al., an increase in the concentration of Cu, Fe, Mn, and Zn in the hair of children living in a region free from oil production compared with a polluted area was described [25].

One should note that the right choice of biomarkers is crucial for assessing the impact of contaminants, including metals, on the human body. Hair samples are widely used to assess human exposure to various pollutants due to their many advantages, such as ease of sample collection and transportation, convenient storage, and higher concentrations of elements than in other bioindicators [26]. Hair deposits trace elements compared to urine, saliva, tear fluid, and blood. Also, hair provides a more accurate determination of trace elements and can reflect quantitative changes in many elements and related metabolic processes over a long period [27]. The correlation described by some authors between the concentration of elements in hair and their level in the body in a physiological or pathological state confirmed the usefulness of hair as a diagnostic tool [8,28]. Thus, there is a vast amount of literature on the adverse effects of heavy metals on human beings’ health.

However, little is known about human exposure to heavy metals in western Kazakhstan’s oil and gas production and processing areas. Only a few studies are available on the essential trace elements in residents living in those areas. This circumstance calls for the need to describe the trace element status of the population living around the fields.

The present study is the first on essential elements in scalp hair of residents from the Caspian region of western Kazakhstan, living near oil and gas fields. There have been no studies comparing the content of essential elements in residents’ hair depending on the distance to the oil and gas fields in the Caspian region of western Kazakhstan.

Hence, the study’s objective is to evaluate the relationship between the concentration of essential trace elements in scalp hair of the western Kazakhstan adult population and the remoteness of their residence from oil and gas fields.

## 2. Materials and Methods

### 2.1. Study Design and Sites Description

We carried out a cross-sectional study during research trips to Aktobe, Mangystau, and West Kazakhstan provinces (oblasts) to determine the trace element status of the adult population. The West Kazakhstan Medical University IRB approved the research (meeting No. 5 dated 13 May 2020), which was performed following the STROBE checklist and the principles of the Helsinki Declaration.

Description of the sites with and without oil and gas production.

Western Kazakhstan is the country’s largest oil and gas producing region, located between Eastern Europe and Central Asia. It consists of four provinces (oblasts): Atyrau, West Kazakhstan, Aktobe, and Mangystau. There is a coastline of considerable length, formed by the coast of the Caspian Sea, washing the Mangyshlak peninsula and the Caspian lowland. 

The Caspian region includes three oil and gas basins: Pre-Caspian, Ustyurt-Bozashi, and Mangyshlak. Oil production volumes reach up to 100–120 million tons per year. At the same time, oil production is expanding due to the giant and large Paleozoic and Mesozoic fields of the Caspian region (Tengiz, Karachaganak, Kashagan and Zhanazhol groups, Uzen, Kalamkas, Zhetybay, Imashevskoye), geotectonically related to the Caspian basin and the west of the Turan plate. Overall, two hundred thirty-three fields have been discovered in the Caspian region—162 sites in the Caspian basin, 55 in Mangyshlak, and 18 in Ustyurt-Bozashi including 8 in the Northern and Middle Caspian waters [29].

Aktobe province (oblast) is located in the northwestern part of Kazakhstan. The annual precipitation ranges from 125 to 300 mm. Aktobe is a large industrial region of Kazakhstan. The main industrial sectors are mining, chemical industry, and ferrous metallurgy. The tense environmental situation in the region is associated with the activities of chromium mining and processing companies and oil and gas facilities. A stable natural-technogenic boron-chromium geochemical province has formed in the area [30]. The West Kazakhstan province is located northwest and occupies 151.34 thousand sq. km. The climate is sharply continental. The annual rainfall ranges from 250 up to 400 mm. The oil and gas industry (Karachaganak and Chinarevskoye fields) and engineering enterprises negatively impact the environment [31]. Mangistau province is located in southwest Kazakhstan, toward the east of the Caspian Sea on the Mangyshlak plateau. The region’s territory constitutes the wormwood-saline desert, extremely arid, with rainfall of about 100–150 mm per year. The Mangistau oblast is a developed industrial region where 25% of Kazakhstan’s oil is produced. The main adverse factors are the lack of freshwater, infertile soils, and constant fluctuations in the level of the Caspian Sea [32].

### 2.2. Sampling

We used a cluster sample for this cross-sectional study. In each cluster, we formed a group with a random sample of recruited volunteers aged 18–60, permanently residing in the study area. A total of 850 individuals, men 350 (41.2%), and women 500 (58.8%), were included in the study. The surveyed population involved in the study came from 32 settlements in western Kazakhstan. These settlements, their distance from the oil fields, and the number of participants in each are enlisted in Table S1 (Supplementary Materials). 

Exclusion criteria were the following: acute infectious, surgical, or traumatic diseases, decompensated chronic somatic diseases, having metal implants (including amalgam fillings), following a vegetarian diet, intake of vitamin and mineral supplements, pregnancy, childbirth less than one year ago, and breastfeeding.

**Figure 1:**
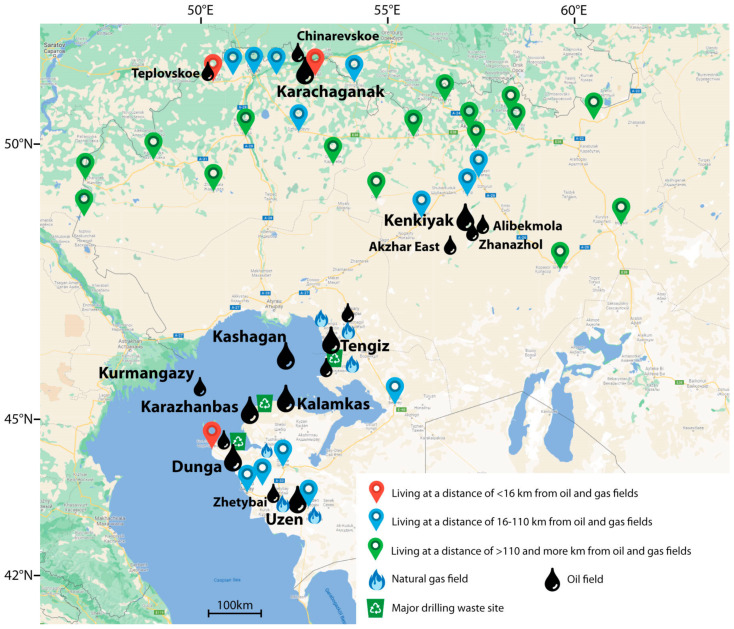
Map of the surveyed areas.

The spatial distribution of the locations is reflected in Figure 1.

We compared the content of trace elements in the hair of residents living near the oil and gas fields fewer than 16 km, from 16 to 110, and more than 110 km, respectively. A distance of up to 16 km is considered the distance at which the consequences of the operation of the oil and gas facilities can affect the human body [33]. When comparing the content of trace elements, for the results of those living out of 110 km, we assumed them to be relatively uncontaminated [34]. Distances from the settlements to fields were calculated using Google Maps. For each location, we applied the coordinates taken from Wikipedia.

Each participant was enrolled after clearly explaining the research goals, the method used to analyze the collected samples, and how her/his identity and privacy would have been protected. The informed consent forms were provided in the Kazakh and Russian languages, of the participant’s choice. These forms are available on reasonable request.

### 2.3. Data Collection

We collected samples from November 2020 to February 2021. Hair samples of at least 0.1 g were obtained by cutting with clean stainless steel scissors from three to five areas at the back of the head [35,36]. In the case of long hair, only the proximal terminus was collected. In these hair samples, we estimated the content of essential trace elements: Co, Cu, Fe, I, Mn, Se, and Zn.

The elemental composition of hair we studied using inductively coupled plasma mass spectrometry (ICP MS) on a NexION 300D mass spectrometer (PerkinElmer Inc., Shelton, CT, USA) equipped with an ESISC-2 DX4 automatic dispenser (Elemental Scientific Inc., Omaha, NE, USA). The ICP-MS operating parameters are shown in Table S2, while the limits of detection (LoD), limits of quantification (LoQ), and background equivalent concentration (BEC) are presented in Table S3.

Hair samples were subjected to preparation by washing and microwave decomposition. The hair strands were washed with acetone, rinsed three times with deionized water, and dried in air at a temperature of 60 °C. After preliminary preparation and taking of weighted amounts (no less than 10 mg) of each hair sample, bio substrates were transferred into chemically stable Teflon tubes with high purity nitric acid.

Microwave digestion was carried out for 20 min at a temperature of 170–180 °C in a “Berghof Speedwave 4 system” (Berghof Products + Instruments, GmbH, 72800 Eningen, Germany). After cooling and equalizing the pressure in the system, the solutions obtained during decomposition were transferred into tubes, and the volume was adjusted to 15 mL with distilled deionized water. The final solution was used for chemical analysis. The system was calibrated using the Universal Data Acquisition Standards Kit (Perkin Elmer Inc., Shelton, CT, USA). Internal online standardization was performed using a Yttrium-89 isotope solution obtained from Yttrium (Y) “Pure Single Element Standard” (Perkin Elmer Inc., Shelton, CT, USA). A certified human hair standard GBW09101 “Humanhair” issued by the Shanghai Institute of Nuclear Research (China) was used for quality control.

#### Statistical Processing

The distribution of the data concentrations of essential trace elements (Co, Cu, Fe, I, Mn, Se, Zn) in the scalp hair was non-Gaussian. The content of essential trace elements in hair is presented with the geometric means (GM), arithmetic means (AM), median (Me), percentiles (P2.5%; P97.5%), maximum (Max), and minimum (Min) values. Qualitative variables are presented as absolute values and percentages.

We analyzed the differences in the content of essential trace elements in three groups depending on the place of residence (<16 km, 16–110 km, and >110 km) using the Kruskal–Wallis H-test. For a posteriori comparisons for these three groups, a new critical level of *p* < 0.017 was used [37].

Multiple linear regression analysis was applied to assess the relationship between concentrations of essential trace elements in the hair (dependent variable) and the settlement’s remoteness from the oil and gas production site (independent variable). We transformed the data using the natural logarithm Ln(X) to perform the multiple linear regression model. Verification of the distribution of variables after the logarithm was carried out using descriptive statistics and graphical methods. The following covariates self-reported by the participants in the questionnaire were included in the models as covariates to adjust for potential confounding: age (continuous), gender (two categories), body mass index (BMI, continuous), and tobacco smoking (two categories).

For statistical testing hypotheses, the critical significance level p was taken equal to 0.05 with a 95% confidence interval. SPPS.v.25 Modeler (IBM) and Statistica.v.10 (StatSoft) software were used for statistical analysis.

## 3. Results

The main features of the study participants are presented in Table S1. The study included n = 850 adults from western Kazakhstan. There were 350 men (41.2%) and 500 (58.8%) women. The average age of men was 42 (31.0; 53.0) years, and women were older by an average of 10 years. The body mass index for men and women was 25.7 (22.5; 28.7) and 25.5 (22.7; 29.1). Among the total sample, smokers were 12.7%, and the city inhabitants were 239 (28.1%). The remoteness of settlements (n = 32) from oil and gas production/processing areas ranged from 2.3 to 475 km (Table S1).

Data on the concentration of essential trace elements in residents’ scalp hair are summarized in Table 1. Data are presented as the arithmetic mean (AM), geometric mean (GM), median (Me), percentiles (P2.5%; 97.5%), and maximum (Max) and minimum (Min) values.

We analyzed comparatively the three groups depending on the distance from oil and gas fields: group 1—less than 16 km; group 2—from 16 to 110 km and group 3—more than 110 km. Findings are presented in Table 2. We observed a significant change in Cu, I, Mn, and Se concentrations. The highest hair concentrations of Cu = 10.96 µg/g, I = 0.391 µg/g, and Mn = 0.559 µg/g have been recorded in residents from the settlements at a distance more than 110 km away from the oil and gas production sites. A posteriori comparisons showed that the content of Cu and I in their hair was significantly higher than that of residents whose settlements belonged to groups 1 and 2 (*p* < 0.001). The content of Mn = 0.361 µg/g in the hair of residents living less than 16 km from the oil and gas production sites was significantly less than that of groups 2 and 3. Concerning Se, we observed the opposite effect. Its concentration decreased with the distance from the fields to 0.540 µg/g; 0.509 µg/g; 0.471 µg/g, respectively, and reliably differed in all three studied groups.

The next step constituted a multiple linear regression analysis (Table 3). We introduced the essential trace elements’ concentrations natural algorithm into the analysis as a dependent variable. The distance from the place of residence to oil and gas production facilities, age, gender, BMI, and smoking were introduced as independent variables. We found that regardless of age, gender, BMI, and smoking, with the distance from the oil and gas production locations (every 100 km), the concentration of Cu and I in residents’ hair increased. An inverse relationship was observed for Co, Fe, Se, and Zn, but for Mn, a direct one. Nonetheless, statistical significance for these trace elements was not found.

## 4. Discussion

Overall, we established a significant difference between the three groups of examined residents of the Caspian region in the concentrations of Cu, I, Se (*p* < 0.001), and Mn (*p* = 0.002). The level of Cu, I, and Mn in the hair of residents living in the closest proximity to active oil and gas fields (<16 km) appeared to be significantly lower. On the contrary, the distribution of Se in residents’ hair demonstrates the opposite trend, reaching a maximum near the working oil and gas facilities (Table 1).

When reporting the elemental status of residents in oil-producing regions, attention is usually focused on the groups of toxic and potentially toxic elements associated with these elements’ pollution of the oil-producing area [38,39,40]. The existing data on the content of essential elements in the hair of residents from oil-producing regions are not enough to perform a comparative analysis. The change in the range of essential elements in the body accompanied by changes in their level in hair can be affected by various factors. The story of essential elements in the body can be influenced by the natural geochemical features of terrains where the population lives and by pollution of the territory of various origins (natural, industrial). In addition, the trace element status reflects multiple biochemical and physiological processes, including those associated with antagonistic or synergistic interactions between elements belonging to both the same and different groups. There are conflicting data on the hair content of manganese, which belongs to the group of essential elements due to its participation in critical physiological processes. At the same time, it is one of the chemical elements associated with the pollution of oil and gas production areas. Some studies confirm the contamination of soils in oil and gas production areas with manganese [18,41], and some report no change in manganese concentration in soils of such regions. [17,42] Studies report an increase in the concentration of Mn in the hair of children living under conditions of industrial pollution [43] and the hair and urine of pregnant women from the regions of gas production by fracturing method [23]. The study by Moon et al. is of great interest as being one of the oldest research for determining the concentration of chemical elements in the hair of residents from oil-producing regions [25]. The authors found a rise in the concentration of manganese, along with other elements from the group of essential ones—iron and zinc, as well as the concentration of calcium and magnesium in the hair of children living in an area classified as unpolluted. In our study, manganese concentration is significantly lower in residents’ hair from the area of oil fields (Table 2).

On the one hand, this can indirectly confirm the absence of significant contamination of this area with manganese compounds. On the other hand, a higher concentration of manganese in the hair of residents of zones remote from the oil and gas facilities (Table 2) could likely be associated with large industrial cities in western Kazakhstan. The economic potential of these cities is formed precisely by ferrous and non-ferrous metallurgy, resulting in the active entry of ecologically dangerous substances into the environment. These substances include recognized pollutants As, Hd, Cd, Be, and heavy metals, classified as essential trace elements—Cu, Fe, Zn, Co, and Mn according to the physiological classification. Albeit the study by Baubekova et al. does not classify western Kazakhstan as a region with a high level of industrial pollution of Fe, Zn, Co, and Mn [44].

When evaluating the content of essential trace elements in the hair of residents of western Kazakhstan, we should realize that the entire territory of settlement belongs to the zone of dry steppes, semi-deserts, and deserts, forming a biogeochemical zone characterized by an excess of zinc and boron and a deficiency of copper, iodine, and cobalt [45].

The decrease in the level of copper is most pronounced in the group of residents from zones located near oil and gas fields (<16 km) (Table 2, Figure 1). Researchers from Poland obtained similar findings in a study conducted in industrially polluted areas. Nowak et al. found a reduced concentration of copper in biological substrates, including hair, among residents of contaminated terrains. The authors attribute the decrease in copper to an increase in the concentration of lead and cadmium in the environment and in the examined individuals’ bodies. As known, these heavy metals belong to the toxic group and are antagonists of copper [46].

There are few papers on iodine content in the biological substrates of the inhabitants of petrochemical provinces. Kudabayeva et al. reported a decrease in iodine concentration in the children’s hair in western Kazakhstan. However, the authors have not focused on the terrain zoning according to the distance of settlements from oil and gas fields [47,48,49]. In the Aral Sea region adjacent to western Kazakhstan, a decline in the iodine in the biological substrates of residents has also been established [50]. The frequency of thyroid diseases associated with decreased function is relatively high in Kazakhstan west, primarily because the region is classified as an iodine-deficient biogeochemical province. Considering that oil and gas fields are located in rural areas, the diet of inhabitants of the settlements around the fields may differ from the diet of urban residents, and a lessening of iodine in their hair may be a consequence of a reduction of iodine intake from food. It has been found that iodine deficiency is common for terrains where the population consumes local agricultural products grown on soils poor in iodine. [51,52]. Formal studies to determine the dietary intake of iodine in western Kazakhstan have not been performed yet. The decrease in hair iodine concentration, probably reflecting the decline in iodine content in the bodies of the population living near oil and gas fields, may result from iodine interactions with antagonists, particularly with Pb, which is a recognized environmental pollutant in oil production [17,34]. Our study also found a decrease in the concentration of Cu in the scalp hair of the oil production zone inhabitants. It is known that copper deficiency can reduce the absorption of iodine by the body and its utilization by the thyroid gland [53,54].

Notably, the regression analysis confirmed the increase in selenium concentration in residents’ hair from the area most affected by the oil and gas industry (Table 1 and Table 2). However, the studied region (western Kazakhstan) is not among the biogeochemical provinces characterized by increased selenium content. Researchers have identified and described selenium-bearing areas in many parts of the world: the eastern part of Russia [55], China [56], Taiwan [57], Punjab [58], and Amazonia [59]. Their studies have assessed the relationship between chronic exposure to environmental selenium and human health. Human health can be affected by abnormally low and excessively high levels of selenium in the environment—in soil, food of local origin, and drinking water [60].

Some authors suppose that selenium levels in the blood and hair are the best biomarkers of exposure because the content of selenium in the blood serum and the hair is strongly correlated. [58]. However, Kousa et al. could not establish such a correlation [61]. According to some researchers, Se in urine is the best tool for determining Se status in the body [62]. Although, it is commonly accepted that selenium, a chemical analog of sulfur, accumulates in nails and hair, as the sulfur-containing amino acids cysteine and methionine comprise hair and nail. This property of selenium is believed to entail a negative effect on the human body, associated with a violation of the functions of proteins due to the replacement of sulfur in amino acids [63,64]. An increase in selenium content in hair can be noted in many pathological conditions. For example, Tinkov et al. found that the Se content in the hair of obese individuals significantly exceeded the control values [65].

However, in the present study, the increased level of selenium in the hair of residents from the oil and gas production zone within 0–16 km, compared with those living in zones at a distance of 16 to 110 km and more than 110 km, did not go beyond the reference values established by the laboratory (Table 1 and Table 4). It may be associated with increased dietary intake of selenium-containing foods such as beef [56], fish [57], or white bread [55]. Some sources evidence a slight increase in the content of selenium in food (meat, dairy products, wheat) in the neighboring region of the Russian Federation [55]. The results of comparing the range of essential trace elements Co, Cu, Fe, I, Mn, Se, and Zn in the hair of residents living in areas with varying degrees of remoteness from pollution sources associated with the operation of oil-and-gas-producing/processing facilities, we confirmed through the multiple linear regression analysis. According to our findings, the increased concentration of Cu and I in hair is associated with increased distance from oil and gas fields. But for Se, the unadjusted regression analysis model describes an inverse relationship (Table 3). When conducting a regression analysis, the food intake and environment are mostly believed to be leading factors influencing the concentration of essential elements in hair. However, additional endogenous and exogenous reasons may play a significant role. Linear regression models created considering confounding factors such as age, gender, BMI, and smoking, confirmed the above patterns described for Cu and I (Table 2).

Table 4 presents comparative results on the hair concentration of essential trace elements Co, Cu, Fe, I, Mn, Se, and Zn, obtained in different populations. These data indicate a significant difference in the distribution of essential elements due to the residence of people in territories belonging to various biogeochemical provinces, characterized by an excess or deficiency of certain elements.

**Figure 2:**
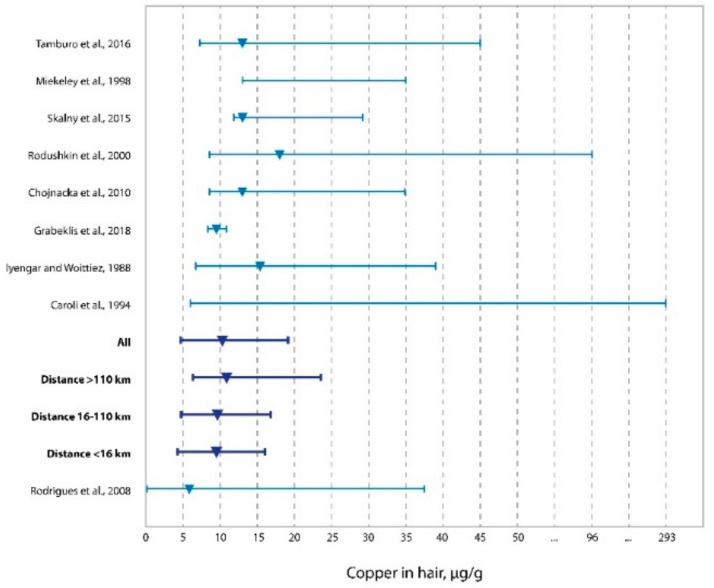
Copper concentrations in residents’ hair from the three zones in western Kazakhstan vs. literature data (medians and ranges) [66,67,68,69,71,72,73,74].

In Figure 2, we presented the distribution of copper concentrations in hair of the adult population of western Kazakhstan living in areas of different distances from oil and gas production zones compared to similar data on the child population of the same area [67], as well as the populations of Italy (Sicily) [74], Brazil [8], Russia [66], Sweden [72], Poland [73], and Canada [70]. This graph also includes data from the research by Iyengar et al. and Caroli et al., with accumulated findings from several studies [68,69]. Of the presented populations, the exposed ones included the population of children from Sicily living in the region of industrial pollution. The rest of the populations were not exposed or had both the exposed and the unexposed individuals (Iyengar et al., Caroli et al.). Noteworthy is the decrease in the median (10.35 µg/g) compared to the rest of the populations (Figure 2), except for a study also performed in western Kazakhstan, where the concentration of copper in children’s hair was found to be 9.5 µg/g.

The decrease in the level of copper in hair is most pronounced in the zone of oil and gas production (<16 km), and as moving away from the sources of pollution, the median concentrations of copper at the distances of 16–110 km and >110 km increase significantly. Despite this, copper concentrations in the inhabitants’ hair from terrains most remote from oil and gas facilities, such as the entire population of western Kazakhstan, remain significantly low.

Supposing changes in copper concentration in the hair are associated with the oil industry is convincing, given the distance difference between the three zones. Yet, the decrease in the copper level in the population of western Kazakhstan as a whole, especially compared to other populations, needs additional rationale. Likely, this phenomenon also suggests reducing the copper concentration related to the biogeochemical province of a zone of dry steppes, deserts, and semi-deserts, to which this region belongs.

**Figure 3:**
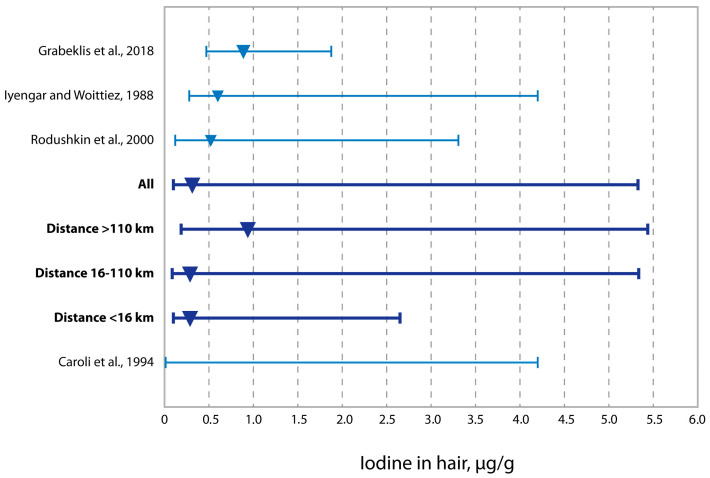
Iodine concentrations in residents’ hair from the three zones in western Kazakhstan vs. literature data (medians and ranges) [67,68,69,72].

Figure 3 shows that the median distribution of iodine concentration in adults’ hair in western Kazakhstan, described in this work, is significantly lower than the same values in the child population from the same region [67], the study by Iyengar et al. [69] and Sweden research [72]. There is also a significant difference in the level of hair iodine depending on the remoteness of oil and gas production enterprises. The hair concentration of iodine is minimal in the zone <16 km and gradually increases, taking the values typical for most inhabitants living at a distance of >110 km from the oil production area.

The present study has some limitations. Heterogeneity of age and gender distribution within and between settlements was the main limitation of the study. Most men were more likely to have short or seldom hair, which was inconvenient for collecting samples. Besides this, many were reluctant to participate in the study for unspoken reasons. As for women, they were more willing to be enrolled in the study. The study format did not imply receiving complete information about the content of trace elements in the environment of western Kazakhstan (soil, water, air). Furthermore, dietary habits also influence trace element status, but we could not scope these habits within the research presented. Although some studies show that while the idea of measuring trace elements in hair is attractive, hair is not a suitable biomarker for assessing I, Cu, and Mn deficiency. In addition, residents of the Atyrau region of the western part of Kazakhstan are not included.

Studies solely focused on recording changes in the level of essential elements in hair toward a decrease or an increase cannot explain the nature of these metamorphoses. Therefore, significant fluctuations in the concentration of essential elements in residents’ hair from the studied region should become the basis for more comprehensive research involving influencing factors as far as possible. The protocol of further examination should include blood tests and determining the amount of trace elements ingested with food. It was also necessary to analyze blood and urine samples to compare and confirm f toxic effects.

Notwithstanding, our data at least confirmed a change in the content of essential elements depending on the distance to/from oil and gas producing facilities.

Significant changes in the concentration level of essential elements in the hair of residents of the studied region should be the basis for additional research, including analysis of the content of toxic, potentially toxic trace elements, antagonistic, and synergistic interactions between them. In future research, it is important to study the content of essential elements in the air, water, and soil of the considered region of Kazakhstan and evaluate their relationship with adverse health effects on people living in the region of oil and gas production in western Kazakhstan.

## 5. Conclusions

In the inhabitants’ hair from the oil and gas production region in western Kazakhstan, the concentrations of Cu and I are significantly interrelated with the distance to oil and gas fields (approaching them). In zones with varying degrees of remoteness from oil and gas fields, we revealed a reduction in Cu, I, and Mn content at a distance of 0–16 km near such sites. The Se concentration decreased with distance from the fields. The data obtained indicate the possible impact of pollutants associated with the activities of oil and gas producing/processing enterprises on a shortage of essential trace elements and consequent health risks.

## Figures and Tables

**Table 1:** Essential trace elements in hair of western Kazakhstan residents (µg/g).

Element	AM	GM	Min	Max	Me (Q1; Q3),	P2.5; P97.5
Co	0.055	0.017	0.001	3.94	0.014 (0.007; 0.031)	0.014 (0.003; 0.361)
Cu	10.64	10.03	2.39	50.86	10.35 (8.56; 12.04)	10.35 (4.71; 19.18)
Fe	26.02	21.58	5.77	204.79	20.14 (14.24; 29.89)	20.14 (8.96; 83.43)
I	0.959	0.388	0.072	67.58	0.315 (0.198; 0.591)	0.315 (0.101; 5.34)
Mn	1.43	0.615	0.074	40.97	0.526 (0.260; 1.127)	0.526 (0.127; 10.94)
Se	0.507	0.472	0.101	9.11	0.496 (0.415; 0.568)	0.496 (0.184; 0.768)
Zn	208.09	190.57	53.08	1200.42	185.59 (152.28; 232.06)	185.59 (85.96; 509.41)

**Table 2:** Concentration of essential trace elements (µg/g) in residents’ hair in three groups depending on the remoteness of the oil and gas production/processing sites.

Element	Distance <16 km (n = 79)				Distance 16–110 km (n = 422)				Distance >110 km (n = 349)				*p* K-W
	AM	GM	Me (Q1; Q3)	P2.5; P97.5	AM	GM	Me (Q1; Q3),	P2.5; P97.5	AM	GM	Me (Q1; Q3),	P2.5; P97.5	
Co	0.037	0.016	0.015 (0.008; 0.027)	(0.004; 0.222)	0.056	0.018	0.015 (0.008; 0.036)	(0.003; 0.361)	0.057	0.016	0.013 (0.007; 0.025)	(0.002; 0.556)	0.076
Cu	9.67	9.20	9.51 ^c^ (8.19; 11.44)	(4.34; 16.01)	9.83	9.33	9.56 ^c^ (7.67; 11.52)	(4.70; 16.98)	11.83	11.13	10.96 ^a,b^ (9.67; 12.78)	(6.15; 23.68)	<0.001
Fe	25.85	20.998	18.53 (13.22; 30.73)	(9.29; 81.38)	26.73	22.07	20.51 (14.58; 30.81)	(8.95; 86.55)	25.20	21.12	20.01 (14.24; 28.44)	(8.93; 76.78)	0.488
I	1.11	0.324	0.238 ^c^ (0.178; 0.480)	(0.100; 2.64)	0.919	0.346	0.290 ^c^ (0.175; 0.543)	(0.095; 5.34)	0.973	0.464	0.391 ^a,b^ (0.250; 0.682)	(0.129; 5.35)	<0.001
Mn	1.14	0.414	0.361 ^b,c^ (0.178; 0.773)	(0.109; 6.88)	1.75	0.669	0.536 ^a^ (0.270; 1.19)	(0.133; 13.35)	1.10	0.608	0.559 ^a^ (0.261; 1.17)	(0.127; 5.22)	0.002
Se	0.545	0.533	0.540 ^b*,c^ (0.466; 0.613)	(0.300; 0.858)	0.530	0.481	0.509 ^a*,c^ (0.433; 0.575)	(0.146; 0.742)	0.470	0.449	0.471 ^a,b^ (0.393; 0.549)	(0.187; 0.771)	<0.001
Zn	207.78	192.69	182.57 (152.18; 42.71)	(93.03; 480.67)	210.21	194.24	190.97 (155.77; 232.06)	(85.63; 490.50)	205.59	185.75	178.87 (149.15; 228.35)	(79.38; 552.48)	0.092

AM—arithmetic mean; GM—geometric mean Mann–Whitney test; differences at the level of *p* < 0.001; ^a^—<16 km; ^b^—16–110 km; ^c^—>110 km; *p* = 0.009 for ^a*^, and ^b*^.

**Table 3:** Crude and adjusted differences in hair concentrations of essential elements.

Element	Crude Difference	95% CI	*p*	Model 1	95% CI	*p*	Model 2	95% CI	*p*	Model 3	95% CI	*p*
Co	0.040	−0.120; 0.040	0.329	−0.044	−0.125; 0.037	0.287	−0.044	−0.126; 0.037	0.282	−0.044	−0.125; 0.038	0.293
Cu	0.074	0.053; 0.096	<0.001	0.073	0.051; 0.095	<0.001	0.072	0.050; 0.094	<0.001	0.072	0.050; 0.094	<0.001
Fe	−0.012	−0.049; 0.025	0.526	−0.012	−0.047; 0.024	0.530	−0.013	−0.049; 0.023	0.023	−0.013	−0.049; 0.023	0.474
I	0.141	0.078; 0.205	<0.001	0.124	0.061; 0.187	<0.001	0.120	0.057; 0.183	<0.001	0.121	0.058; 0.185	<0.001
Mn	0.016	−0.057; 0.089	0.667	0.015	−0.059; 0.089	0.689	0.011	−0.063; 0.085	0.772	0.010	−0.064; 0.084	0.799
Se	−0.032	−0.055; −0.009	0.007	−0.022	−0.045; 0.001	0.058	−0.022	−0.045; 0.000	0.055	−0.023	−0.045; 0.000	0.054
Zn	−0.023	−0.049; 0.003	0.085	−0.012	−0.038; 0.014	0.360	−0.016	−0.041; 0.010	0.227	−0.015	−0.041; 0.010	0.236

Model 0: Adjusted for distance; Model 1: Adjusted for age and gender; Model 2: As in model 1 + BMI; Model 3: As in model 2 + Smoking.

**Table 4:** Summary of published data on concentrations of essential trace elements (µg/g) in hair in different populations.

Sample Type & Location	Co Me (Range)	Cu Me (Range)	Fe Me (Range)	I Me (Range)	Mn Me (Range)	Se Me (Range)	Zn Me (Range)	References
Present study, n = 850	0.014 (0.003; 0.361)	10.35 (4.71; 19.18)	20.14 (8.96; 83.43)	0.315 (0.101; 5.34)	0.526 (0.127; 10.94)	0.496 (0.184; 0.768)	185.59 (85.96; 509.41)	
Occupationally non-exposed Russian adult population, n = 7256	0.015 (0.009; 0.073)	13.0 (11.8; 29.2)	12.5 (9.6; 31.5)	-	0.52 (0.29; 1.76)	0.296 (0.093; 0.482)	186.4 (134.7; 301.9)	Skalny et al., 2015 [66]
Children aged 7–11 years from Kazakhstan, n = 836	0.018 (0.012; 0.027)	9.5 (8.3;10.9)	22.8 (16.2; 32.1)	0.886 (0.465; 1.871)	0.852 (0.513; 1.408)	0.388 (0.283; 0.471)	121 (81;164)	Grabeklis et al., 2018 [67]
Children and adults selected from various countries	0.07; 1.70	6; 293	10; 900	0.03; 4.2	0.04; 24.00	0.002; 6.600	53.7; 327.0	Caroli et al., 1994 [68]
Adult population selected from various countries: nearly 100, 000 individuals from 55 countries	0.077 (0.0004; 0.500)	16.4 (6.8; 39.0)	33 (13;177)	0.60 (0.27; 4.20)	1.2 (0.2; 4.4)	0.53 (0.20; 1.40)	175 (124; 320)	Iyengar and Woittiez, 1988 [69]
Canada, adults, ng/mg	0.023 (0.004; 0.140)	20.3 (9.0; 61.3)	-	-	0.067 (0.016; 0.570)	0.54 (0.37; 1.37)	162 (129; 209)	Gulle et al., 2005 [70]
Brazil, adults, n = 1091	(0.26;0.47)	(13;35)	(6.0;15)	-	(0.26;0.75)	(0.38;0.7)	(125; 165)	Miekeley et al., 1998 [71]
Sweden, children+adults, from 1 year old up to 76, n = 114	0.010 (0.002; 0.063)	18 (8.5; 96.0)	8.4 (4.9; 23.0)	0.52 (0.13; 3.31)	0.35 (0.08; 2.41)	0.79 (0.48; 1,84)	144 (68; 198)	Rodushkin et al., 2000 [72]
Brazil, adult healthy population n = 280	-	5.90 (0.02; 37.60)	-	-	0.70 (0.05; 6.70)	-	-	Rodrigues et al., 2008 [8]
Poland, Wroclaw, students aged 20, n = 117	0.789 (0.775; 0.985)	13.00 (8.51; 34.97)	22.1 (16.9; 29.6)	-	0.627 (0.459; 1.046)	-	181 (140; 371)	Chojnacka et al., 2010 [73]
Sicily, children 11–14 years old, n = 943	0.040 (0.003; 0.450)	13.0 (7.1; 45.0)	-	-	0.31 (0.01; 1.60)	0.50 (0.13; 1.40)	200 (110; 295)	Tamburo et al., 2016 [74]

## Data Availability

Data are available from the corresponding author upon request.

## Supplementary Materials

The following supporting information can be downloaded at: https://www.mdpi.com/article/10.3390/toxics10070364/s1, Table S1: Brief description of the study sites and population; Table S2: System characteristics and settings for trace element analysis; Table S3: Limits of detection (LoD), limits of quantification (LoQ), background equivalent concentration (BEC).

## References

[1] Chanturidze T., Adams O., Tokezhanov B., Naylor M., Richardson E. (2015). Building policy-making capacity in the Ministry of Health: The Kazakhstan experience. Hum. Resour. Health.

[2] Gulis G., Aringazina A., Sangilbayeva Z., Zhan K., de Leeuw E., Allegrante J.P. (2021). Population Health Status of the Republic of Kazakhstan: Trends and Implications for Public Health Policy. Int. J. Environ. Res. Public Health.

[3] Webb E., Moon J., Dyrszka L., Rodriguez B., Cox C., Patisaul H., Bushkin S., London E. (2018). Neurodevelopmental and neurological effects of chemicals associated with unconventional oil and natural gas operations and their potential effects on infants and children. Rev. Environ. Health.

[4] Radelyuk I., Tussupova K., Persson M., Zhapargazinova K., Yelubay M. (2021). Assessment of groundwater safety surrounding contaminated water storage sites using multivariate statistical analysis and Heckman selection model: A case study of Kazakhstan. Environ. Geochem. Health.

[5] Alimbaev T., Yermagambetova K., Kabyltayeva S., Issayev A., Kairat Z., Mazhitova Z. (2020). Environmental problems of the oil and gas industry in Kazakhstan. E3S Web Conf..

[6] Rovira J., Nadal M., Schuhmacher M., Domingo J.L. (2021). Environmental impact and human health risks of air pollutants near a large chemical/petrochemical complex: Case study in Tarragona, Spain. Sci. Total Environ..

[7] Benhaddya M.L., Boukhelkhal A., Halis Y., Hadjel M. (2016). Human Health Risks Associated with Metals from Urban Soil and Road Dust in an Oilfield Area of Southeastern Algeria. Arch. Environ. Contam. Toxicol..

[8] Rodrigues J.L., Batista B.L., Nunes J.A., Passos C.J., Barbosa F. (2008). Evaluation of the use of human hair for biomonitoring the deficiency of essential and exposure to toxic elements. Sci. Total Environ..

[9] Brázdová Z.D., Pomerleau J., Fiala J., Vorlová L., Müllerová D. (2014). Heavy Metals in Hair Samples: A Pilot Study of Anaemic Children in Kazakhstan, Kyrgyzstan and Uzbekistan. Cent. Eur. J. Public Health.

[10] Domingo J.L., Marquès M., Nadal M., Schuhmacher M. (2020). Health risks for the population living near petrochemical industrial complexes. 1. Cancer risks: A review of the scientific literature. Environ. Res..

[11] Marquès M., Domingo J.L., Nadal M., Schuhmacher M. (2020). Health risks for the population living near petrochemical industrial complexes. 2. Adverse health outcomes other than cancer. Sci. Total Environ..

[12] Chiang T.-Y., Yuan T.-H., Shie R.-H., Chen C.-F., Chan C.-C. (2016). Increased incidence of allergic rhinitis, bronchitis and asthma, in children living near a petrochemical complex with SO2 pollution. Environ. Int..

[13] Rovira E., Cuadras A., Aguilar X., Esteban L., Santos A.B., Zock J.-P., Sunyer J. (2014). Asthma, respiratory symptoms and lung function in children living near a petrochemical site. Environ. Res..

[14] Rafiee A., Delgado-Saborit J.M., Aquilina N.J., Amiri H., Hoseini M. (2021). Assessing oxidative stress resulting from environmental exposure to metals (Oids) in a middle Eastern population. Environ. Geochem. Health.

[15] Rafiee A., Delgado-Saborit J.M., Sly P.D., Quémerais B., Hashemi F., Akbari S., Hoseini M. (2020). Environmental chronic exposure to metals and effects on attention and executive function in the general population. Sci. Total Environ..

[16] Bussan D., Harris A., Douvris C. (2019). Monitoring of selected trace elements in sediments of heavily industrialized areas in Calcasieu Parish, Louisiana, United States by inductively coupled plasma-optical emission spectroscopy (ICP-OES). Microchem. J..

[17] Relić D., Sakan S., Anđelković I., Popović A., Đorđević D. (2019). Pollution and Health Risk Assessments of Potentially Toxic Elements in Soil and Sediment Samples in a Petrochemical Industry and Surrounding Area. Molecules.

[18] Dore M.P., Farias C., Hamacher C. (2017). Offshore drilling effects in Brazilian SE marine sediments: A meta-analytical approach. Environ. Monit. Assess..

[19] Rezende C., Lacerda L., Ovalle A., Souza C., Gobo A., Santos D. (2002). The effect of an oil drilling operation on the trace metal concentrations in offshore bottom sediments of the Campos Basin oil field, SE Brazil. Mar. Pollut. Bull..

[20] Breuer E., Stevenson A., Howe J., Carroll J., Shimmield G. (2004). Drill cutting accumulations in the Northern and Central North Sea: A review of environmental interactions and chemical fate. Mar. Pollut. Bull..

[21] Varrica D., Tamburo E., Alaimo M.G. (2021). Levels of trace elements in human hair samples of adolescents living near petrochemical plants. Environ. Geochem. Health.

[22] Varrica D., Tamburo E., Milia N., Vallascas E., Cortimiglia V., De Giudici G., Dongarrà G., Sanna E., Monna F., Losno R. (2014). Metals and metalloids in hair samples of children living near the abandoned mine sites of Sulcis-Inglesiente (Sardinia, Italy). Environ. Res..

[23] Caron-Beaudoin É., Bouchard M., Wendling G., Barroso A., Bouchard M.F., Ayotte P., Frohlich K.L., Verner M.-A. (2019). Urinary and hair concentrations of trace metals in pregnant women from Northeastern British Columbia, Canada: A pilot study. J. Expo. Sci. Environ. Epidemiol..

[24] Skalny A.V., Kaminskaya G.A., Krekesheva T.I., Abikenova S.K., Skalnaya M.G., Berezkina E.S., Grabeklis A.R., Tinkov A.A. (2017). The level of toxic and essential trace elements in hair of petrochemical workers involved in different technological processes. Environ. Sci. Pollut. Res..

[25] Moon J., Smith T.J., Tamaro S., Enarson D., Fadl S., Davison A.J., Weldon L. (1986). Trace metals in scalp hair of children and adults in three Alberta indian villages. Sci. Total Environ..

[26] Esteban M., Castaño A. (2009). Non-invasive matrices in human biomonitoring: A review. Environ. Int..

[27] Molina-Villalba I., Lacasaña M., Rodríguez-Barranco M., Hernández A.F., Gonzalez-Alzaga B., Aguilar-Garduño C., Gil F. (2015). Biomonitoring of arsenic, cadmium, lead, manganese and mercury in urine and hair of children living near mining and industrial areas. Chemosphere.

[28] Gil F., Hernández A.F., Márquez C., Femia P., Olmedo P., López-Guarnido O., Pla A. (2011). Biomonitorization of cadmium, chromium, manganese, nickel and lead in whole blood, urine, axillary hair and saliva in an occupationally exposed population. Sci. Total Environ..

[29] Azhgaliev D.K., Karimov S.G., Isaev A.A. (2018). Regional study is the next important stage in evaluation of oil and gas industry potential of sedimentary basins of Western Kazakhstan. Georesursy.

[30] Batyrova G., Tlegenova Z., Umarova G., Kononets V., Umarov Y., Kudabayeva K., Aitmaganbet P., Amanzholkyzy A. (2021). Microelement Status of the Adult Population in Western Kazakhstan. Hum. Ecol..

[31] Kenessaryiev U.I., Yerzhanova A.E., Kenessary D.U., Kenessary A.U. (2016). Trends of change in demographic indices of population in the area of oil and gas deposits of the republic of Kazakhstan. Gig. Sanit..

[32] Sakieva K.Z., Mamyrbaev A.A. (2016). State of health of the population of the one out of oil and gas extraction regions of Kazakhstan. Gig. Sanit..

[33] McKenzie L.M., Crooks J., Peel J.L., Blair B.D., Brindley S., Allshouse W.B., Malin S., Adgate J. (2019). Relationships between indicators of cardiovascular disease and intensity of oil and natural gas activity in Northeastern Colorado. Environ. Res..

[34] Pragst F., Stieglitz K., Runge H., Runow K.-D., Quig D., Osborne R., Runge C., Ariki J. (2017). High concentrations of lead and barium in hair of the rural population caused by water pollution in the Thar Jath oilfields in South Sudan. Forensic Sci. Int..

[35] Pozebon D., Scheffler G., Dressler V.L. (2017). Elemental hair analysis: A review of procedures and applications. Anal. Chim. Acta.

[36] Szynkowska M.I., Marcinek M., Pawlaczyk A., Albińska J. (2015). Human hair analysis in relation to similar environmental and occupational exposure. Environ. Toxicol. Pharmacol..

[37] Chan Y., Walmsley R.P. (1997). Learning and Understanding the Kruskal-Wallis One-Way Analysis-of-Variance-by-Ranks Test for Differences Among Three or More Independent Groups. Phys. Ther..

[38] González N., Esplugas R., Marquès M., Domingo J.L. (2021). Concentrations of arsenic and vanadium in environmental and biological samples collected in the neighborhood of petrochemical industries: A review of the scientific literature. Sci. Total Environ..

[39] Yuan T.-H., Chio C.-P., Shie R.-H., Pien W.-H., Chan C.-C. (2016). The distance-to-source trend in vanadium and arsenic exposures for residents living near a petrochemical complex. J. Expo. Sci. Environ. Epidemiol..

[40] Anticona C., Bergdahl I., Lundh T., Alegre Y., Sebastian M.S. (2011). Lead exposure in indigenous communities of the Amazon basin, Peru. Int. J. Hyg. Environ. Health.

[41] Breuer E., Shimmield G., Peppe O. (2008). Assessment of metal concentrations found within a North Sea drill cuttings pile. Mar. Pollut. Bull..

[42] Nadal M., Mari M., Schuhmacher M., Domingo J.L. (2009). Multi-compartmental environmental surveillance of a petrochemical area: Levels of micropollutants. Environ. Int..

[43] Dongarrà G., Varrica D., Tamburo E., D’Andrea D. (2012). Trace elements in scalp hair of children living in differing environmental contexts in Sicily (Italy). Environ. Toxicol. Pharmacol..

[44] Baubekova A., Akindykova A., Mamirova A., Dumat C., Jurjanz S. (2021). Evaluation of environmental contamination by toxic trace elements in Kazakhstan based on reviews of available scientific data. Environ. Sci. Pollut. Res. Int..

[45] Schlesinger W.H., Bernhardt E.S. (2013). Biogeochemistry: An Analysis of Global Change.

[46] Nowak B., Chmielnicka J. (2000). Relationship of Lead and Cadmium to Essential Elements in Hair, Teeth, and Nails of Environmentally Exposed People. Ecotoxicol. Environ. Saf..

[47] Kudabayeva K.I., Batyrova G.A., Bazargaliyev Y.S., Baspakova A.M., Sakhanova S.K. (2018). Hair trace element composition in 6-to 12-year-old children with goiter in West Kazakhstan, a province of the Republic of Kazakhstan. J. Elementol..

[48] Kudabayeva K., Batyrova G., Bazargaliyev Y., Agzamova R., Nuftieva A. (2017). Microelement status in children with enlarged thyroid gland in West Kazakhstan region. Georgian Med. News.

[49] Kudabayeva K.I., Koshmaganbetova G.K., Mickuviene N., Skalnaya M.G., Tinkov A.A., Skalny A.V. (2016). Hair Trace Elements are Associated with Increased Thyroid Volume in Schoolchildren with Goiter. Biol. Trace Elem. Res..

[50] Namazbayeva Z.I., Berzhanova R.S., Ulzhibayeva R.R., Iskendirova A.Z., Kyzkenova A.Z., Mahmetova A.M. (2015). Microelement profile of Aral region adult population. Med. Tr. Promyshlennaia Ekol..

[51] Zimmermann M.B. (2009). Iodine deficiency. Endocr. Rev..

[52] Ahmad S., Bailey E.H., Arshad M., Ahmed S., Watts M.J., Young S.D. (2021). Multiple geochemical factors may cause iodine and selenium deficiency in Gilgit-Baltistan, Pakistan. Environ. Geochem. Health.

[53] Turan E., Turksoy V.A. (2021). Selenium, Zinc, and Copper Status in Euthyroid Nodular Goiter: A Cross-Sectional Study. Int. J. Prev. Med..

[54] Kravchenko V.I., Andrusyshyna I.M., Luzanchuk I.A., Polumbryk M.O., Tarashchenko Y.M. (2020). Association Between Thyroid Hormone Status and Trace Elements in Serum of Patients with Nodular Goiter. Biol. Trace Elem. Res..

[55] Skalny A., Burtseva T.I., Salnikova E.V., Ajsuvakova O.P., Skalnaya M.G., Kirichuk A., Tinkov A.A. (2019). Geographic variation of environmental, food, and human hair selenium content in an industrial region of Russia. Environ. Res..

[56] Li M., Yun H., Huang J., Wang J., Wu W., Guo R., Wang L. (2020). Hair Selenium Content in Middle-Aged and Elderly Chinese Population. Biol. Trace Elem. Res..

[57] Skalny A.V., Skalnaya M.G., Serebryansky E.P., Zhegalova I.V., Grabeklis A.R., Skalnaya O.A., Skalnaya A.A., Huang P.-T., Wu C.-C., Bykov A.T. (2018). Comparative Hair Trace Element Profile in the Population of Sakhalin and Taiwan Pacific Islands. Biol. Trace Elem. Res..

[58] Chawla R., Filippini T., Loomba R., Cilloni S., Dhillon K.S., Vinceti M. (2020). Exposure to a high selenium environment in Punjab, India: Biomarkers and health conditions. Sci. Total Environ..

[59] Rocha A.V., Cardoso B.R., Cominetti C., Bueno R.B., de Bortoli M.C., Farias L.A., Favaro D.I.T., Camargo L.M.A., Cozzolino S.M.F. (2014). Selenium status and hair mercury levels in riverine children from Rondônia, Amazonia. Nutrition.

[60] Vinceti M., Filippini T., Cilloni S., Bargellini A., Vergoni A.V., Tsatsakis A., Ferrante M. (2017). Health risk assessment of environmental selenium: Emerging evidence and challenges. Mol. Med. Rep..

[61] Kousa A., Loukola-Ruskeeniemi K., Hatakka T., Kantola M. (2021). High manganese and nickel concentrations in human hair and well water and low calcium concentration in blood serum in a pristine area with sulphide-rich bedrock. Environ. Geochem. Health.

[62] Takahashi K., Suzuki N., Ogra Y. (2018). Effect of administration route and dose on metabolism of nine bioselenocompounds. J. Trace Elem. Med. Biol..

[63] Jablonska E., Vinceti M. (2015). Selenium and Human Health: Witnessing a Copernican Revolution?. J. Environ. Sci. Health Part C Environ. Carcinog. Ecotoxicol. Rev..

[64] Hatfield D.L., Tsuji P.A., Carlson B.A., Gladyshev V.N. (2014). Selenium and selenocysteine: Roles in cancer, health, and development. Trends Biochem. Sci..

[65] Tinkov A.A., Skalnaya M.G., Ajsuvakova O.P., Serebryansky E.P., Chao J.C.-J., Aschner M., Skalny A.V. (2021). Selenium, Zinc, Chromium, and Vanadium Levels in Serum, Hair, and Urine Samples of Obese Adults Assessed by Inductively Coupled Plasma Mass Spectrometry. Biol. Trace Elem. Res..

[66] Skalny A., Skalnaya M., Tinkov A.A., Serebryansky E.P., Demidov V.A., Lobanova Y.N., Grabeklis A., Berezkina E.S., Gryazeva I.V., Skalny A. (2015). Hair concentration of essential trace elements in adult non-exposed Russian population. Environ. Monit. Assess..

[67] Grabeklis A.R., Abazov K.A., Skalny A.A., Lobanova Y.N. (2018). Regional approach to providing WFP un services: Comparison of multielement hair data of schoolchildren from Tajikistan, Azerbaijan, Kazakhstan, Turkmenistan, Bangladesh, Macedonia, Croatia, and Russian federation. Microelem. Med..

[68] Caroli S., Alimonti A., Coni E., Petrucci F., Senofonte O., Violante N. (1994). The Assessment of Reference Values for Elements in Human Biological Tissues and Fluids: A Systematic Review. Crit. Rev. Anal. Chem..

[69] Iyengar V., Woittiez J. (1988). Trace elements in human clinical specimens: Evaluation of literature data to identify reference values. Clin. Chem..

[70] Goullé J.P., Mahieu L., Castermant J., Neveu N., Bonneau L., Lainé G., Bouige D., Lacroix C. (2005). Metal and metalloid multi-elementary ICP-MS validation in whole blood, plasma, urine and hair: Reference values. Forensic Sci. Int..

[71] Miekeley N., Carneiro M.T.D., Portodasilveira C. (1998). How reliable are human hair reference intervals for trace elements?. Sci. Total Environ..

[72] Rodushkin I., Axelsson M.D. (2000). Application of double focusing sector field ICP-MS for multielemental characterization of human hair and nails. Part II. A study of the inhabitants of northern Sweden. Sci. Total Environ..

[73] Chojnacka K., Zielińska A., Górecka H., Dobrzański Z., Górecki H. (2010). Reference values for hair minerals of Polish students. Environ. Toxicol. Pharmacol..

[74] Tamburo E., Varrica D., Dongarrà G. (2016). Gender as a key factor in trace metal and metalloid content of human scalp hair. A multi-site study. Sci. Total Environ..

