# Supplementary material for: Essential Trace Elements in Scalp Hair of Residents across the Caspian Oil and Gas Region of Kazakhstan
Source: Toxics. 2022 Jun 30;10(7):364. doi: 10.3390/toxics10070364 (PMC9317645; doi:10.3390/toxics10070364)
Supplement: Supplementary file 1 [file toxics-10-00364-s001.zip › toxics-1738929-supplementary.pdf]

Table S1. Brief description of the study sites and population.

Settlements	Distance from the Oil Field	n (%)	Age, Me (Q1; Q3), years	Men, n (%)	BMI, Me (Q1;Q3), kg/m²	Smokers, n (%)
Distance <16 km						
Aksay	11 km from the giant Karashyganak field	27(3.18)	36.0(26.0;47.0)	11(40.7)	25.3(23.0;35.2)	6(22.2)
Fort-Shevchenko	2.3 km from the Bautino field, and10 km from the drilling oil waste disposal site	29(3.41)	38.0(32.0;44.0)	13(44.8)	26.8(25.0;31.1)	6(20.7)
Taskala	5 km from the Teplovskoye	23(2.71)	56.0(44.0;60.0)	12(52.2)	26.0(24.7;29.6)	5(21.7)
Distance 16-110 km						
Aktau (city of regional importance)	50 km from the Dunga field	61(7.18)	41.0(32.0;46.0)	31(50.8)	25.2(22.5;28.0)	14(23.0)
Beyneu	96 km from the Shagyrly-Shomyshty field	55(6.47)	41.0(34.0;52,0)	32(58.2)	26.9(23.2;30.1)	8(14.5)
Chingirlau	76 km from the Karashyganak	10(1.18)	32.5(24.0;50.0)	3(30.0)	24.8(20.8;27.3)	1(10.0)
Fedorovka	72 km from the Karashyganak field	9(1.06)	31.0(26.0;44.0)	3(33.3)	23.4(19.5;26.2)	1(11.1)
Zhanaozen	20 km from the Uzen field	38(4.47)	41.0(29.0;49.0)	19(50.0)	26.3(23.5;29.7)	4(10.5)
Karaulkeldy	75 km from the Akzhar Vostochny field	28(3.29)	44.0(33.0;55.0)	12(42.9)	27.0(22.9;29.3)	4(14.3)
Mangyshlak (village)	62 km from the Dunga field	18(2.12)	37.0(25.0;48.0)	15(83.3)	25.6(23.1;27.5)	4(22.2)
Mugojar	108 km from the Alibek Mola field	17(2.00)	46.0(41.0;50.0)	4(23.5)	24.9(22.9;26.6)	2,0(11.8)
Peremetnoe	40 km from the Teplovskoye field	23(2.71)	31.0(26.0;39.0)	8(34.8)	22.3(20.5;28.7)	2(8.7)
Temir	91 km from the Zhanazhol field	17(2.00)	46.0(32.0;54.0)	4(23.5)	24.5(23.7;26.7)	2(11.8)
Uralsk (city of regional importance)	40 km from the Chinarevskoye field	72(8.47)	36.5(30.5;49.5)	25(34.7)	25.3(21.7;28.4)	6(8.3)
Shetpe	78 km from the Zhetybay field	52(6.12)	38.5(28.0;50.5)	25(48.1)	25.7(23.6;29.4)	9(17.3)
Distance >110km						
Aitekebi	319 km from the Kenkiyak field	15(1.76)	45.0(34.0;57.0)	2(13.3)	25.3(23.8;27.4)	-
Aktobe (city of regional importance)	196 km from the Alibek Mola field	106(12.47)	45.5(35.0;58.0)	36(34.0)	25.3(22.8;28.5)	9(8.5)
Akzhayik	165 km from the Karashyganak	22(2.59)	52.0(33.0;57.0)	11(50.0)	26.8(21.5;29.7)	2(9.1)
Alga	152 km from the Alibek Mola	20(2.35)	42.5(35.5;51.5)	2(10.0)	24.3(22.1;27.4)	1(5.0)
Batamsha	376 km from the Karashyganak	14(1.65)	47.0(26.0;60.0)	4(28.6)	27.6(23.1;29.3)	1(7.1)
Khromtau	197 km from the Alibek Mola	22(2.59)	45.0(34.0;50.0)	8(36.4)	24.1(21.5;26.9)	5(22.7)
Irgiz	305 km from the Kenkiyak	13(1.53)	46.0(41.0;56.0)	2(15.4)	25.9(24.7;27.8)	-
Kobda	219 km from the Karashyganak	15(1.76)	49.0(29.0;58.0)	5(33.3)	28.2(23.1;31.2)	4(26.7)

Martyk	251 km from the Karashyganak	28(3.29)	44.0(32.0;53.5)	12(42.9)	28.1(24.7; 29.6)	4(14.3)
Zhangala	286 km from the Karashyganak	11(1.29)	39.0(27.0;50.0)	5(45.5)	26.8(19.6;29.1)	-
Zhanibek	475 km from the Karashyganak	12(1.41)	45.5(34.5;53.5)	4(33.3)	23.5(23.2;25.3)	-
Zhimpity	101 km from the Karashyganak	22(2.59)	33.0(22.0;48.0)	15(68.2)	24.3(20.9;27.5)	1(4.5)
Kaztalovka	187 km from the Teplovskoye	12(1.41)	45.5(35.0;57.0)	3(25.0)	26.2(24.2;28.9)	-
Karatobe	166 km from the Karashyganak	10(1.18)	41.0(30.0;45.0)	2(20.0)	23.9(22.2;27.5)	-
Saykhin	358 km from Teplovskoye	10(1.18)	41.0(28.0;57.0)	3(30.0)	27.8(23.5;31.2)	2(20.0)
Shalkar	205 km from the Kenkiyak field	15(1.76)	54.0(43.0;60.0)	2(13.3)	23.5(21.8;26.9)	1(6.7)
Oyil	259 km from the Karashyganak	24(2.89)	42.5(26.0;53.5)	17(70.8)	26.9(22.1;29.7)	4(16.7)
Men		350(41.2)	35.0(27.0;51.0)	-	25.7(22.5;28.7)	104(29.7)
Women		500(58.8)	45.0(35.0;54.0)	-	25.5(22.7;29.1)	4(0.8)
Total		850(100)	42.0(31.0;53.0)	310(43.1)	25.6(22.6;29.0)	108(12.7)

Table S2. System characteristics and settings for trace element analysis.

**Plasma power, W**	**1500**
Plasma argon flow, l/min	18
Aux argon flow, l/min	1.6
Nebuliser argon flow, l/min	0.98
Sample introduction system	ESI ST PFA concentric nebuliser and ESI PFA cyclonic spray chamber (Elemental Scientific Inc., Omaha, NE, USA)
Sampler and skimmer cone material	Platinum
Injector	ESI Quartz 2.0 mm I.D.
Sample flow, µl/min	637
Internal standard flow, µl/min	84
Dwell time and acquisition mode	10–100 ms and peak hopping for all analytes
Sweeps per reading	1
Reading per replicate	10
Replicate number	3

Table S3. Limits of detection (LoD), limits of quantification (LoQ), background equivalent concentration (BEC)

**Element**	**LoD, ppb**	**BEC, ppb**	**LoQ, ppb**
Co	0.0013	0.0021	0.0039
Cu	0.0035	0.0235	0.0105
Fe	0.0791	0.11	0.2373
Mn	0.0102	0.03	0.0306
Se	0.0129	0.026	0.0387
Zn	0.0709	0.22	0.2127
I	0.0075	0.012	0.0225

LoD – limit of detection, LoQ - limits of quantification, BEC - background equivalent concentration, ppb – part per billion
